# Clinicopathologic Characteristics of Typical Medullary Breast Carcinoma: A Retrospective Study of 117 Cases

**DOI:** 10.1371/journal.pone.0111493

**Published:** 2014-11-06

**Authors:** Zhaohui Chu, Hao Lin, Xiaohua Liang, Ruofan Huang, Qiong Zhan, Jingwei Jiang, Xinli Zhou

**Affiliations:** 1 Department of Oncology, Huashan Hospital, Fudan University, Shanghai, China, 200040; 2 Department of Oncology, Shanghai Medical College, Fudan University, Shanghai, China, 200032; Stavanger University Hospital, Norway

## Abstract

**Purpose:**

This study analyzed the clinicopathologic characteristics of typical medullary breast carcinoma (TMBC) in a cohort of Chinese patients.

**Methods:**

We conducted a retrospective review of clinical data including general information, pathologic results, treatment regimens, and patient survival in cases of TMBC diagnosed between February 2004 and April 2011.

**Results:**

A total of 117 patients were enrolled, with a median age of 52 years (range, 28∼92 years). Stage I and II disease accounted for 31.6% and 61.6% of the cases, respectively. Hormonal receptor negative disease (estrogen receptor negative, 68.4%; progestogen receptor negative, 86.3%) was more prevalent in this population. Human epidermal growth factor receptor-2 (HER-2) positivity was 20.5%, while equivocal and HER-2 negative cases represented 16.2% and 63.2% of the cohort. The triple-negative, luminal, and HER-2 overexpressing subtypes constituted 44.4%, 31.6%, and 15.4% of the cases, respectively. The various TMBC subtypes showed no differences regarding tumor size, rates of lymph node(s) metastasis, TNM staging, treatment regimens, and 2-year recurrence rates. However, patients with triple-negative disease were more likely to be younger, when compared to those with luminal disease (P = 0.002). At a median follow-up of 56 months (range, 2–112 months), the 2-year disease-free survival and overall survival rates were 99.1% and 98.2%, respectively.

**Conclusion:**

Early stage disease dominated the study cohort, and at two years after surgery, recurrence was extremely low. The heterogeneity of molecular subtypes was clearly shown, and no apparent differences were found among the clinicopathologic characteristics of the triple-negative, luminal, and HER-2 overexpressing subtypes.

## Introduction

Medullary breast carcinoma (MBC) is one of the invasive breast carcinoma subtypes, which was first precisely defined by Ridolfi et al in 1977 [1]. MBC can be divided into general categories of typical medullary breast carcinoma (TMBC), and atypical medullary breast carcinoma (AMBC) based on the following five criteria: predominantly (>75%) syncytial growth pattern, microscopically completely circumscribed, moderate to marked diffuse mononuclear stromal infiltrate, absence of microglandular features and intraductal components, and moderate or marked nuclear pleomorphism [1]. A diagnosis of TMBC requires that all five of the above criteria are satisfied, while AMBC deviates slightly from TMBC, by requiring that all of the criteria except one (>75% syncytial growth pattern) are satisfied. MBC accounts for <5%of invasive breast cancers, and has a more favorable prognosis than invasive ductal carcinoma (IDC) [1]–[6]. However, recent study found that 95% of the MBCs belonged to basal-like phenotype (negative for estrogen receptor (ER), progesterone receptor (PR), and human epidermal growth factor receptor-2 (HER-2)) based on a gene expression analysis [7]. This is interesting, because a previous report stated that a basal-like phenotype is associated with a worse prognosis [1]. Moreover, some studies have reported that the medullary subtype comprises up to 30%∼40% of ER and PR positive tumors, as well as ∼10% of HER-2 overexpressing tumors [6]–[13]. These findings are in contrast with the histopathological characteristics of the basal-like phenotype. In the current study, we examined the clinicopathologic characteristics of MBC in a local population, with the goal of further optimizing the treatment decisions made for Chinese patients. To reduce the heterogeneity of the study, we focused only on TMBC patients.

## Materials and Methods

### Ethics

The protocol for this study was approved by the Institutional Review Board of Huashan Hospital, Fudan University. Written informed consent was given by participants for their clinical records to be used in this study. All patient data were anonymized and de-identified in a confidential manner. The information in the data set was used exclusively for the purpose of this study, and was not shared with other individuals or organizations.

### Study Design

**Figure 1:**
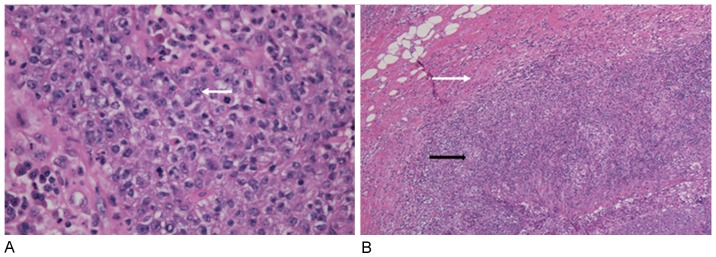
Histopathological features of TMBC. (A) Typical syncytial growth pattern of TMBC, with high-grade nuclear atypia (white arrow); ×400. (B) Pushing margin of TMBC (white arrow) and dense lymphoplasmacytic infiltration (black arrow); ×200.

We conducted a retrospective review of clinical data obtained from patients diagnosed with TMBC between February 2004 and April 2011 at Huashan Hospital, Fudan University (Shanghai, China). The data inclusion criterion was that the histopathological features of the disease agreed with those described in the 2003 WHO classification for TMBC (consistent with the Ridolfi's criteria) by showing the following features: syncytial architecture in >75% of the tumor mass, a predominant and dense lymphoplasmacytic infiltrate, histological circumscription or pushing margins, high-grade nuclear atypia, and the absence of a glandular or tubular structure [14]. The pathologic changes in a typical case which satisfied these criteria are shown in Figure 1.

### Pathology analysis

ER positive (ER+) and PR positive (PR+) tumors were defined as tumors having >1% of their cells showing the appropriate nuclear staining. Hormonal receptor positive tumor was defined as one showing ER+ and/or PR+ staining, and HER-2 status was evaluated per ASCO/CAP consensus [15]. IHC staining of 3+ (uniform, intense membrane staining of >30% of invasive tumor cells) was considered as HER-2 overexpression/amplification. IHC staining of 0 or 1+ was considered to indicate a lack of HER-2 expression. Molecular subtypes were classified as luminal subtype (ER+ and/or PR+, HER-2 IHC 0∼3+), HER-2 overexpressing subtype (ER- and PR-, HER-2 IHC 3+), triple-negative subtype (ER- and PR-, HER-2 IHC 0∼1+), or unknown (ER- and PR-, HER-2 IHC 2+), based on guidelines established at the St. Gallen Consensus 2011 [16]. CK5/6 positivity was defined as tumors having >1% of their cells showing the appropriate cytoplasmic and/or membranous staining.

Histopathology and immunohistochemistry results were reviewed by two experienced pathologists. Disagreements were resolved by discussions with a third expert.

### Survival

Cases selected for our review had received follow-up at 3 to 6-month intervals during the first two years, 6 to 12 month intervals during the next three to five years, and annually thereafter. During these follow-up visits, patients received both a clinical examination and an imaging evaluation. Disease-free survival (DFS) was defined as the period between the first surgery and the recurrence of TMBC. Overall survival (OS) was defined as the period between the first surgery and death or last follow-up. The last follow-up for a patient in our study was conducted on July 1, 2013.

### Statistical analysis

Statistical analyses were performed using SPSS Statistics for Windows, Version 17.0. Chicago, IL: SPSS Inc. The inter-rater agreement between two pathologists was analyzed with kappa test. Differences in patient lymph node status, TNM staging, and recurrence rates among the four subtypes of TMBC were analyzed using Fisher's exact test. Differences in patient age and tumor size among the four subtypes were analyzed by Analysis of Variance. P-values for post hoc multiple comparisons were computed by LSD method. The crude survival rate was calculated using the direct method with the following equation: 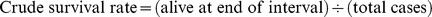



## Results

### General information

A total of 117 patients diagnosed with TMBC between February 2004 and April 2011 were enrolled in this study. All patients were female, and the median age was 52 years (range, 28–92 years). The study included a slightly higher proportion of premenopausal (55.6%) than post-menopausal women (44.4%). No patient had ever received hormonal replacement treatment, and none were pregnant or lactating at the time of diagnosis. Seven patients (6.0%) reported a family history of breast cancer. All patients had initially presented with a breast lump, and were admitted after a median period of 15 days (range, 2–1460 days), during which the mass had enlarged in 11 (9.4%) patients. Pain of the mass presented in 25 patients, without correlation with menstrual cycle. Nipple discharge was found in 2 cases. A solitary mass was detected in most of the cases, while one patient presented with 2 lesions in different quadrants of ipsilateral breast; both lesions were later found to be medullary breast carcinoma. There was no difference between bilateral breasts regarding the incidence of carcinoma. One patient presented with bilateral breast cancer, and a final pathology report indicated medullary carcinoma and IDC for each side.

### Pathology

Stage I, II, and III disease accounted for 31.6%, 61.6%, and 6.8% of the cases, respectively, and the median tumor size was 2.5 cm (range, 1–7.0 cm). Thirty-one (26.5%) cases were confirmed with lymph node metastasis, as determined by axillary lymph nodes dissection. Five elderly patients with no lymph node enlargement detected before surgeries were not given axillary lymph node dissection due to their age. ER- diseases were more prevalent in the cohort, accounting for 68.4% of cases. A similar result was found regarding PR status, with 86.3% patients presenting PR- diseases. Immunohistochemistry results showed HER-2 3+ in 24 (20.5%) cases, 2+ in 19 (16.2%) cases, 1+ in 37 (31.6%) cases, and 0 in 37 (31.6%) cases. CK5/6 positivity was 55.7% (29/52) among triple-negative subtypes. The distribution of molecular subtypes is shown in Table 1. The overall concordance between the two pathologists was 96.7%, and almost perfect agreement was achieved in evaluating the molecular subtypes (kappa value of 0.817).

**Table 1:** Distribution of molecular subtypes in TMBC patients.

Molecular subtypes	ER	PR	HER-2 IHC	Case(s)	Total N (%)
Luminal	+	+	0	6	37(31.6%)
	+	+	1+	5	
	+	+	2+	2	
	+	+	3+	3	
	+	−	0	4	
	+	−	1+	7	
	+	−	2+	7	
	+	−	3+	3	
Triple-negative	−	−	0	27	52 (44.4%)
	−	−	1+	25	
HER-2 overexpression	−	−	3+	18	18 (15.4%)
Unknown	−	−	2+	10	10 (8.5%)

Abbreviation: TMBC, typical medullary breast cancer; ER, estrogen receptor; PR, progesterone receptor; HER-2 IHC, human epidermal growth factor receptor-2immunohistochemistry.

### Treatment arrangement

Modified radical mastectomy dominated over surgical options in the cohort, with 90 (76.9%) patients engaged in this approach. Lumpectomy and radical mastectomy contributed to only 12.8% and 10.3% of the cases, respectively. Chemotherapy regimens were assigned in accordance with NCCN guidelines for IDC treatment. With the exception of seven patients aged >75 years, all patients had received adjuvant chemotherapy. The most often used chemo regimen was a combination of epirubicin, cyclophosphamide and fluorouracil, followed by a combination of epirubicin, cyclophosphamide and paclitaxel/docetaxel. The designated regimens were administered per NCCN guidelines, and adjusted according to individual situations; they were not based on different molecular subtypes. All patients completed 6 cycles chemotherapy without serious side effects. Hormonal therapy for HR positive patients was initiated after adjuvant chemotherapy had been completed. Patients who presented with a tumor >5 cm and/or a positive lymph node(s), and patients who underwent lumpectomy were given adjuvant radiotherapy within 6 months after surgery. Only four of the 24 patients with HER-2 3+ disease received adjuvant Herceptin therapy, which was given every three weeks. All four patients who underwent target therapy ceased treatment within 3 to 5 months due to financial reasons.

### Follow-up data

With 4 patients lost, the median follow-up time was 56 (range, 2–112) months. One patient died of heart disease 2 months after surgery, and cancer recurred in only 2 other patients, both of whom were triple-negative disease, stage IIA (T_2_N_0_M_0_). DFS times for these 2 cases were 98 and 11 months, respectively. The former patient is still alive as of today (111 months survival), while the latter patient died at 24 months after surgery due to disease relapse. A total of 113 patients were followed for >2 years, and that group showed DFS and OS rates of 99.1% and 98.2%, respectively.

### Clinicopathologic presentation of different subtypes

The clinicopathologic characteristics of the different subtypes of TMBC and their prognosis are shown in Table 2. The treatments used for different TMBC subtypes are shown in Table 3. There was no apparent difference in the distribution of subtypes treated with surgery and adjuvant chemotherapy. However, adjuvant radiotherapy was used more often for the management of triple-negative subtype than for HER-2 overexpressing and luminal subtypes. The various TMBC subtypes showed no differences regarding tumor size, rates of lymph node(s) metastasis, TNM staging. However, patients with triple-negative disease were more likely to be younger, when compared to those with luminal disease (P = 0.002).

**Table 2:** Clinicopathologic characteristics of different molecular subtypes in TMBC patients.

	Luminal (n = 37)	Triple-negative (n = 52)	HER-2 overexpression (n = 18)	Unknown (n = 10)	P
Age*, years (mean ± S.D.)	57.11±13.06	49.56±10.34	53.06±10.77	55.50±8.95	0.019
Tumor size, cm (mean ± S.D.)	2.47±0.75	2.66±1.12	3.00±0.87	2.39±1.00	0.231
T≤2 cm	15 (40.5%)	23 (44.2%)	5 (27.8%)	6 (60.0%)	0.553
2<T≤5 cm	22 (59.5%)	28 (53.8%)	13 (72.2%)	4 (40.0%)	
T>5 cm	0 (0.0%)	1 (1.90%)	0 (0.0%)	0 (0.0%)	
Lymph node status, n (%)					0.557
N0	29 (78.4%)	38 (73.1%)	14 (77.8%)	5 (50.0%)	
N1	6 (16.2%)	9 (17.3%)	3 (16.7%)	4 (40.0%)	
N2	1 (2.7%)	4 (7.7%)	0 (0.0%)	1 (10.0%)	
N3	1 (2.7%)	1 (1.9%)	1 (5.6%)	0 (0.0%)	
TNM, n (%)					0.997
I	12 (32.4%)	17 (32.7%)	5 (27.8%)	3 (30.0%)	
IIA	17 (45.9%)	25 (48.1%)	8 (44.4%)	6 (60.0%)	
IIB	5 (13.5%)	7 (13.5%)	3 (16.7%)	1 (10.0%)	
III	3 (8.1%)	3 (5.8%)	2 (11.1%)	0 (0.0%)	
Recurrence in 2-year, n (%)	0 (0.0%)	2 (3.8%)	0 (0.0%)	0 (0.0%)	0.716

*Difference exists between subgroups; Luminal vs. Triple-negative, MD (Mean Difference)  = 7.55, P = 0.002. Luminal vs. HER-2 overexpression, MD = 4.05, P = 0.212. Luminal vs. Unknown, MD = 1.61, P = 0.689. Triple-negative vs. HER-2 overexpression, MD = −3.50, P = 0.258. Triple-negative vs. Unknown, MD = −5.94, P = 0.129. HER-2 overexpression vs. Unknown, MD = −2.44, P = 0.583.

**Table 3:** Treatment of patients with different molecular subtypes of TMBC.

	Luminal (n = 37)	Triple-negative (n = 52)	HER-2 overexpression (n = 18)	Unknown (n = 10)
Surgery				
Modified radical mastectomy	31 (83.8%)	37 (71.2%)	14 (77.8%)	8 (80.0%)
Radical mastectomy	2 (5.4%)	5 (9.6%)	3 (16.7%)	2 (20.0%)
Lumpectomy	4 (10.8%)	10 (19.2%)	1 (5.6%)	0
Adjuvant chemotherapy				
Yes	31 (83.8%)	52 (100.0%)	17 (94.4%)	10 (100.0%)
No	6 (16.2%)	0 (0%)	1 (5.6%)	0
Adjuvant radiotherapy				
Yes	13 (35.1%)	20 (38.5%)	3 (16.7%)	6 (60.0%)
No	24 (64.9%)	32 (61.5%)	15 (83.3%)	4 (40.0%)
Hormonal therapy				
Yes	37 (100.0%)	0 (0.0%)	0 (0.0%)	0 (0.0%)
No	0 (0.0%)	0 (0.0%)	0 (0.0%)	0 (0.0%)

## Discussion

In this study, we detected a significantly higher rate of ER+ disease (31.6%) in TMBC patients, compared to such rates observed in Western countries. Huober et al [17] reported that only 19% of enrolled patients showed ER+ carcinoma, and a study with 3,348 MBC patients conducted in the USA [18] described similar low rates of ER and PR positivity (16.3% and 14%, respectively). Based on the St. Gallen International Expert Consensus of 2011, luminal subtypes should include both luminal A (ER+ and/or PR+, HER-2 +/−, and low Ki-67 index) and luminal B (ER+ and/or PR+, HER-2 +/−, and high Ki-67 index) diseases. In our analysis, luminal subtypes represented 31.6% of the TMBC cases, which was similar to results previously reported in another Chinese cohort (42%) [19]. However, these percentages were significantly higher compared to those reported in other studies, in which the MBCs were mostly triple-negative carcinomas and dominated across all the subtypes [7], [20]–[21]. Therefore, ER status may be one of several factors contributing to the difference in long-term survival between Eastern and Western women. MBC has been reported as a distinct subgroup of basal breast cancer, with limited myoepithelial differentiation as shown by gene expression analysis [7]. CK5/6 protein is a member of the basal/myoepithelial cytokeratin family, and is frequently detected in triple-negative invasive breast cancer (60%∼72% positivity rate) [22]–[23]. In the current study, however, only 55% of triple-negative breast cancer cases were positive for CK5/6 protein expression. Further studies investigating other basal/myoepithelial cytokeratin proteins such as CK14, CK17, and EGFR are required to address the above difference in TMBC patients. Flucke et al [24] reported that HER-2 overexpression was more prevalent in MBC than in HR negative high grade IDC (26% vs. 7%). Similarly, disease with HER-2 overexpression represented 15.4% of all TMBC cases in our study cohort, even though some cases (8.5%) had not yet been characterized by FISH. Therefore, we suggest that HER-2 amplification may be a factor contributing to the favorable prognosis of MBC.

The molecular subtype is one of the most important factors impacting the clinical outcome of breast cancer. It has been widely accepted that the triple-negative subtype and HER-2 overexpressing subtype both predict a poor clinical outcome. In this study, patients with triple-negative disease were younger at the time of diagnosis compared to patients with other subtypes. This finding correlates with our previous understanding of triple-negative cancer, and we believe that such patients should benefit from receiving standard chemotherapy and increased surveillance. The TMBC patients in our cohort were clinically managed according to NCCN guidelines for IDC, and no deviations were recommended regarding surgical treatment, adjuvant chemotherapy, hormonal therapy or adjuvant radiotherapy. No apparent difference was found in the distribution of surgery and adjuvant chemotherapy. While on the other hand, the ratio of adjuvant radiotherapy in triple-negative subtype was higher than that in HER-2 overexpressing and luminal subtypes, which may probably due to higher proportion of large tumor (>5 cm), positive lymph nodes and breast conserving therapy in the latter. However, concern still remains for the long-term survival of patients who are treated based on generally accepted guidelines used for invasive cancer, because there is no universally recognized standard of care for TMBC. Xue et al [25] reported that the 2-year DFS rates for triple-negative and HER-2 overexpressing IDC were 79% and 82%, respectively; whereas for our TMBC patients, the 2-year DFS and OS rates were 98.2% and 99.1%, respectively. Our analysis showed a more favorable prognosis for TMBC patients compared to IDC patients during a short follow-up period; however, an analysis over a long-term follow-up period still needs to be completed before general conclusions can be reached. Except for patient age, no significant differences were found regarding the clinicopathologic characteristics of given molecular subtypes, including tumor size, rates of lymph node(s) metastasis, and TNM staging. During the short follow-up period, only two relapses of triple-negative carcinoma were detected; however, it remains to be determined whether variations in molecular subtype will affect long-term prognosis. In our cohort, the median age of patients upon entry into the study was 52 years, which was similar to the age reported for studies conducted in Europe [16]. Early stage disease was more prevalent among all patients, with 93.2% of the cases being stage I or II cancer, and this finding was similar to that in Reinfuss et al's [26] study. We also found that the probability of lymph node(s) metastasis was <30%, which was similar to results in other studies conducted in Europe and the USA [17]–[18]. Eisinger et al [27] reported six cases of MBC (19%) among 32 BRCA1-associated breast cancers, compared to only one MBC (0.5%) among 200 patients without a family history of breast cancer. This comparison suggests an important association between TMBC and a previous family history of breast cancer. However, in our cohort, only 6.0% of the TMBC patients had a family history of breast cancer, indicating that the correlation between TMBC and the BRCA1 mutation remains to be determined. In 2012, Chandrika et al [28] reported a case of triple-negative synchronous bilateral medullary carcinoma, and a similar result was found in one of our patients who presented with two synchronous ipsilateral breast lesions which showed triple-negative disease.

Overall, our designated TMBC patients were mostly diagnosed at early stages. Triple-negative disease was prevalent, but not dominant in the cohort, while heterogeneity was commonly presented. Whether or not molecular subtype is the most important factor affecting TMBC patients' prognosis is yet to be determined. Our evidence suggests the possibility of differences among races, and therefore, genetic analysis may be needed for Chinese TMBC patients. The short-term prognosis for patients in our cohort was favorable; nonetheless, the differences among subtypes need to be investigated in studies with a longer duration.
